# Laparoscopic “Intraperitoneal Underlay Mesh”-Plus: A Viable Approach for Incisional-Ventral Hernia Repair

**DOI:** 10.3389/jaws.2025.14459

**Published:** 2025-06-18

**Authors:** Elie Chelala, Brian Jacob

**Affiliations:** 1 Belgian Section of Abdominal Wall Surgery Chirec/Delta-Hospital, Departement of Digestive & Bariatric Surgery, Brussels, Belgium; 2 International Hernia Collaboration, Icahn School of Medicine at Mount Sinai, NYC Partner, Laparoscopic Surgical Center of New York, New York, NY, United States

**Keywords:** intraperitoneal onlay mesh, ventral hernia repair, laparoscopic IPUM+, augmentation technique, incisional ventral hernia repair

## Abstract

The current Intraperitoneal Underlay Mesh (IPUM), previously referred to as IPOM (onlay), initially faced several challenges due to design and methodological shortcomings, particularly with the use of a bridging technique without defect closure. These limitations contributed to elevated recurrence rates, mesh bulging, seroma formation, and suboptimal abdominal wall function. Although complications such as adhesions, bowel erosion, fistula formation, and mesh migration were rare, they were mostly associated with non-composite or poorly designed meshes and inadequate fixation. These concerns led to growing skepticism regarding intraperitoneal mesh placement and a shift in preference toward retrorectus mesh positioning. Since 2007, the evolution of IPUM+ techniques—where the “+” denotes primary defect closure combined with the use of advanced composite meshes, has led to significantly improved outcomes. Long-term studies, meta-analyses, and randomized trials have demonstrated better functional results, reduced complications, and broader acceptance among surgeons and patients. These advancements have positioned IPUM+ as a reliable and effective option, especially when long-term outcomes of alternative techniques such as eTEP, ventral TAPP, or robotic approaches remain under long term follow up evaluation. IPUM+ continues to serve as a valuable technique for small to moderate or recurrent ventral hernias when performed by experienced surgeons. Future directions should aim to define its role in personalized hernia care, integrating hybrid methods and emerging technologies for complex repairs.

## Introduction

### Historical Context and Evolution of IPUM Techniques

The term “intraperitoneal onlay mesh plus” (IPOM+) is commonly used in the literature; however, there is a growing preference for the term “intraperitoneal underlay mesh plus” (IPUM+), which more accurately reflects the technique, as the mesh is placed in an underlay rather than an onlay position [1]. The early iterations of the intraperitoneal onlay mesh (IPUMs) technique faced substantial challenges due to design and methodological shortcomings, particularly when utilizing a non-closed defect or bridging approach even in large width’ defects. These drawbacks and limitations included high recurrence rates, mesh bulging, seroma formation, and poor abdominal wall function restoration. Much of this was attributed to inappropriate mesh types (e.g., UltraPro, Phasix®, ePTFE), inadequate mesh sizes, and insufficient fixation. These inadequacies often led to complications, albeit rare, such as adhesions, bowel erosion, fistula formation, encapsulation, and mesh migration, especially with non-composite or poorly designed meshes. As a result, skepticism about the safety and efficacy of intraperitoneal mesh placement became widespread [2, 3].

However, since 2007, the advent of IPUM+ techniques—which emphasize defect closure before the reinforcement with composite mesh—has significantly shifted outcomes. Modern materials and methodologies in IPUM+ have addressed the limitations, reduced drawbacks, of earlier designs. Long-term studies and meta-analyses have consistently shown better functional and clinical outcomes, reduced complication rates, and increased acceptance of this approach among surgeons and patients alike. These advancements underscore the importance of defect closure and the use of composite meshes with appropriate fixation in optimizing results [4–8].

## Materials and Methods

### Advancements in the Minimal Invasive Techniques

Recent meta-analyses of minimally invasive surgery (MIS) repairs, encompassing 11 studies with 2,320 patients, show that IPUM+ leads to fewer reoperations and complications when performed meticulously and in accordance with current safety protocols [9].

In contrast to traditional methods, newer techniques like Enhanced View Totally Extraperitoneal (eTEP) have shown higher rates of readmissions, surgical site infections (SSI), seromas, hematomas, and bowel obstructions. However, strong evidence on mid- and long-term outcomes remains limited and requires further validation [9–11].

Among other innovative approaches, the transabdominal preperitoneal (TAPP) technique for ventral hernia repair allows for mesh placement outside the peritoneal cavity, potentially reducing adhesions and complications associated with intraperitoneal meshes. Notably, it preserves fascial structures by avoiding transection.

Robotic TAPP offers several additional advantages:

- Enhanced precision and visualization through 3D imaging and improved dexterity, facilitating complex dissection and suturing.
- Lower complication and readmission rates compared to open repair.
- Shorter hospital stays and quicker recovery.
- Higher fascial closure rates, exceeding 90% versus ∼50% in laparoscopic repair, potentially improving functional outcomes.

However, current short-term data suggest that extraperitoneal mesh placement—whether via robotic or laparoscopic TAPP—is not clearly superior to intraperitoneal mesh for small to moderate ventral hernias [12, 13].

### Reducing Chronic Pain and Mesh-Related Adhesions: A Peak Into the eTEP Technique

The comparative efficacy and safety of IPUM+ and eTEP remain areas of ongoing debate, with studies presenting mixed results. Outside of oftentimes biased opinions, there has been no convincing level 1 evidence that IPUM plus should be abandoned or that newer procedures to get mesh into the extra peritoneal or retrorectus space has benefits that outweigh the IPUM+ or its inherent risks which we will detail below. Quite the contrary actually.

Wieland et al. reported no significant differences in postoperative pain scores (measured by VAS on Day 3) between the two techniques. They concluded that eTEP is a safe and feasible option, potentially offering advantages such as shorter hospital stays. [11]. However, this conclusion appears to be biased due to several factors:

1. Uneven patient distribution: A significantly larger number of eTEP patients were included in the study, skewing the analysis.
2. Unexplained variations in hospital stays: IPUM+ patients with defects under 7 cm had an unexpectedly longer length of stay (4 days) compared to the typical 2-day maximum in other practices.
3. Complication profiles: eTEP patients showed a higher proportion of Clavien-Dindo IIIb complications, alongside an overall higher complication rate for IPUM+ that involved fewer patients.

The “Prove it” randomized prospective trial showed no differences between robotic suturing and laparoscopic tacking for mesh fixation for IPUM+ procedures in reducing pain complaints. [15]. In fact, the robotic approach had a significantly longer operative time compared to the laparoscopic method, which was not offset by any demonstrable clinical benefit.

As well the myth that IPUM+ led to more severe fistula forming adhesions was proven false by Maskal et al in a 2024 review of a large registry database [16] concluding that intraperitoneal mesh for repair of small to medium-sized hernias has an extremely low rate of long-term mesh-related complications. It remains a safe and durable option for hernia surgeons.

Furthermore, multicenter randomized trials like the “Reveal” study comparing eTEP to IPUM+, conducted by Clayton P. et al., highlight the risks associated with robotic eTEP repairs. These studies revealed that avoiding fixation in robotic eTEP did not reveal a benefit in postoperative pain to offset any perceived benefits [17].

The eTEP ventral hernia repair requires the division of the posterior rectus sheath bilaterally. This division of an anatomically important structure comes with potential serious risks, including but not limited to possible permanent abdominal wall bulging from a result of either a rectus muscle denervation injury, or from chronic atrophic rectus muscle changes after the posterior sheath is divided. This permanent abdominal wall change is not to be taken lightly and has led to debates between the technique pioneers on whether or not the posterior sheath should be closed or not. Thus the procedure is still somewhat experimental. This complication is avoided when an IPUM+ is performed or could be by the ventral TAPP approach.

Retrorectus seroma and hematoma are also inherent risks to the eTEP ventral repair, unlikelyseen in the IPUM+ repair. These two complications can lead to posterior sheath breakdown, another serious complication specific to eTEP, which can present as an acute postoperative small bowel obstruction, a delayed recurrence, or as an adhesiogenic process to exposed uncoated mesh, leading to intraabdominal adhesions. Therefore this eTEP ventral procedure does not completely eliminate the risk of potential adhesions in the abdominal cavity.

From a general perspective, attention should be paid to the potential risk of disruption in any defect closure exceeding 7 cm in width, particularly when running sutures are placed under high tension, regardless of the surgical approach—be it laparoscopic eTEP, robotic, laparoscopic ventral TAPP, or even IPUM+. In the context of laparoscopic ventral TAPP, Mitura K. et al. emphasized that to minimize this risk, all peritoneal defects and flaps must be carefully closed, and the dissected flap should be sufficiently large to prevent “tear-outs” caused by excessive tension during flap closure [14].

Many authors have reported higher recurrence rate by 3 times in W4-W5 verses W1-W3, in IPUM+ long term follow up [9, 18–20].

We might certainly be concerned with the limitations of each technique and recommend a tailored approach, according to patient’s characteristics and surgeon’s judgement.

Chronic pain, a key concern for IPUM+, can be effectively minimized with meticulous surgical techniques, including:

- Careful adequate fixation to prevent nerve entrapment,
- Avoidance of mesh wrinkling, and
- Employing augmentation techniques with controlled physiological tension.

When these principles are followed, studies report a chronic pain incidence as low as 2.5%. [18–21]. Additionally, a large cohort study by Chelala et al. [4] involving 733 laparoscopic ventral hernia repairs (LVHR) demonstrates the favorable outcomes of IPUM+, with:

- 47% of patients remaining adhesion-free,
- Only 11% experiencing minor omental adhesions, and
- <3% developing minimal serosal adhesions during mid-term follow-up.

These findings underscore the continued relevance of IPUM+ when executed with modern materials and optimal surgical techniques, particularly for patients with appropriate defect sizes and minimal risk factors.

### Advances in Mesh Technology

Modern composite meshes, atraumatic resorbable tackers or glues in combination to trans facial sutures (TFS), and photoactive polymer fixation devices have significantly improved the outcomes of IPUM+ repairs. These innovations minimise direct contact between the mesh and abdominal contents, drastically reducing adhesion risks. Studies of composite meshes indicate a low risk of complications, with long-term follow-ups showing mesh-related complications in only 1.5% of cases [18–20]. Findings by Beldi, Suwa, and others confirm that proper material selection, mesh fixation, and primary fascial closure under physiological tension are essential for achieving low recurrence rates and minimising mesh-related complications [5, 19, 20].

### Importance of Surgeon Expertise and Technique

Enhanced surgical techniques, including precise tissue handling, defect closure under physiological tension, optional peritoneal sac resection, and appropriate fixation under low intra-abdominal pressure, play a pivotal role in reducing complications. These refined methodologies, combined with advanced composite mesh materials, have established IPUM+ as a distinct and more reliable alternative to earlier intraperitoneal bridging procedures. Adequate fascial defect closure and sufficient lateral mesh overlap effectively restore abdominal wall dynamics, reduce dead space, and minimize recurrence and mesh-related risks [18–23].

## Discussion: Evidence Supporting IPUM+ in Recent Studies

### Clinical Efficacy and Outcomes

Several studies, including those by Del Campo et al., underscore the effectiveness of IPUM+ for ventral hernia repair. These studies report low recurrence rates, enhanced patient satisfaction, and rapid recovery times. Chelala’s analysis of 1,326 cases reinforces IPUM+ as a viable and reliable technique for hernia defects under 8 cm in width, despite the emergence of alternative approaches. Among others, Del Campo et al. compared defect closure to non-closure and found that closure significantly reduced recurrence, reinforcing its importance in achieving optimal outcomes in IPUM+ repairs [18–21, 24].

### Key Studies Highlighting Long-Term Safety and Recurrence Rates

- M. Rosen et al.: Long-term cohort data confirm that IPUM+ is an effective and durable method for small- to moderate-sized hernias, with extremely low recurrence rates and minimal adhesion-related complications when composite meshes are used optimally. The researchers concluded that composite meshes placed intraperitoneally are safe when proper techniques are employed, challenging the notion that IPUM+ should be abandoned [15].
- X. Huang et al.: In a systematic review, Huang concluded that the IPUM+ technique significantly reduces recurrence, seroma formation, and mesh bulging while minimising mesh contact with intestines, thereby reducing adhesion risks. Overall, IPUM+ is considered a safe and effective approach [25].
- Marcolin, Stoikes, and Romanowska, et al.: These studies advocate for combining external defect closure with laparoscopic IPUM+ reinforcement for larger defects. This approach benefits patients by lowering recurrence rates and reducing postoperative complications [24, 26, 27].

### Meta-Analyses and Long-Term Comparisons

- Giuffrida et al. reported long-term results for laparoscopic IPUM, with a median follow-up of 9.3 years. The study found a low recurrence rate of 4.9% in patients with hernias under 5 cm in width. The authors concluded that laparoscopic IPUM may be effectively preferred for smaller hernias, while alternative techniques should be considered for larger defects to reduce complication rates. These findings are consistent with the European Hernia Society (EHS) guidelines [28].
- As reported by Hauters et al., a higher mesh-to-defect area ratio (M/D) is considered a potential predictive factor for lower recurrence rates. Consequently, studies have shown that larger defects repaired using IPUMs were associated with higher recurrence rates and mesh-related complications [29, 30], which has contributed to skepticism regarding the use of intraperitoneal meshes.
- Maskal et al. and Ann Nguyen Tuan et al. reported a reoperation rate of only 0.62% up to 6years follow-up due to mesh-related complications in small- to medium-sized hernias, supporting IPUM+ as a safe and durable option for hernia surgeons [16, 31].
- Petro CC et al. found favorable long-term outcomes for IPUM+ with composite mesh, such as recurrence rates, chronic pain, and quality of life. Such data advocate for the durability and safety of IPUM+ when contrasted to newer techniques like eTEP or robotic-assisted repairs [17].
- Köckerling et al. found no statistically significant differences in pain and recurrence rates between IPUM and open sublay anterior abdominal wall hernia repair. Moreover, they reported no additional complications related to intestinal adhesion to the mesh [32].
- Holihan et al. provided data showing that, when performed adequately, IPUM+ has comparable long-term outcomes to sublay repairs, with fewer infections but slightly higher recurrence rates [33].
- Rasador et al. reported in their recent study on TAPP v/s IPOM, 2025: No differences were seen between both techniques regarding ileus, hernia recurrence, operative time, seroma, small bowel obstruction, vascular injury, and bowel injury. Subgroup analysis for robotic VHR showed similar results. After performing a leave-one-out sensitivity analysis, they obtained a shorter hospital LOS (MD  – 0.56 days; 95% CI  – 0.86,  – 0.25; p < 0.05) and VAS scores within 24 h of surgery (MD  – 1.04; 95% CI  – 1.61,  – 0.47; p < 0.05) for the TAPP technique.

However they did not compare VAS beyond 72 h, where it’s supposed to be equivalent as reported in literature [12].

### Rationale Against the Complete Abandonment of IPUM+

#### Role of IPUM+ in Abdominal Wall Hernia Repairs

IPOM+ remains a highly accessible and effective option for “Swiss-cheese” or recurrent hernias, particularly in patients with prior anterior surgeries or compromised abdominal walls where extraperitoneal techniques may not be feasible. In certain high-risk cases, such as emergency and in morbidly obese patients, IPUM+ offers shorter operative times, a less invasive approach, and quicker recovery.

#### Mesh Location and Complication Rates

While extraperitoneal mesh placement is theoretically advantageous for avoiding adhesions to the mesh, long-term comparative data favoring eTEP or robotic techniques over IPUM+ remain limited. R. Dixit et al. concluded: The available data on Patient-Reported Outcome Measures (PROMs) for laparoscopic and robotic primary ventral and incisional hernia repairs are scarce and highly heterogeneous, making it difficult to assess the superiority of the laparoscopic technique over the robotic technique. Further studies with uniform reporting of PROMs for laparoscopic and robotic primary ventral and incisional hernia repairs are needed [34].

Trylisky and Rasador et al. In 2 different systematic review and metanalysis, on eTEP vs. IPUM+, concluded equal safety profile, and SSO. TEP has longer operative time, with only better early 7 days, post operative pain [35, 36].

### Emphasis on Risk Reduction Strategies

#### Advancements in Mesh Design and Adhesion Prevention

Recent advancements, such as anti-adhesive mesh coatings and absorbable adhesion barriers, have demonstrated effectiveness in significantly reducing adhesion formation and related complications in IPUM+ procedures.

In our experience, following a standardized IPUM+ procedure with long-term follow-up, we performed second-look surgeries on 126 patients (9.5%). Among these, 45.23% were found to be adhesion-free, 42.08% exhibited minor adhesions classified as Müller I, and 12.69% presented with serosal adhesions classified as Müller II. To our knowledge, this low rate of mesh-related complications can be attributed to:

- Appropriate primary physiologic fascial closure using a “U reverse type” technique.
- Exclusive use of trans fascial suturing to secure the composite mesh, later supplemented by resorbable tackers or limited TFS (Trans fascial Sutures).
- Complete preservation of the anti-adhesive barrier in a clean and protected surgical field with great mesh overlap.

These findings strongly support the safe use of composite mesh in the majority of patients, achieving high satisfaction rates and good outcomes in small- to medium-sized hernias. Based on extensive experience and supportive literature, we advocate for the continued use of IPUM+ as a valid option in the tailored approach to laparoscopic ventral hernia repair (LVHR) [4, 6, 18–20, 23, 29].

#### Patient Selection as a Key Factor

Expert input suggests that IPUM+ is contraindicated in the following scenarios:

- Large defects with or without loss of domain (LOD).
- Presence of abdominal skin grafts.
- Need for the removal of sizable prosthetic mesh.
- Active entero-cutaneous fistula.
- Ischemic or gangrenous bowel.
- Contaminated surgical field (e.g., intra-abdominal sepsis or faecal peritonitis).
- Cirrhosis with caput medusae.

Nevertheless, IPUM+ remains suitable and justified for carefully selected patients:

- Small to moderate ventral or incisional hernias with defect widths less than 7 cm, especially in patients with prior anterior abdominal surgeries or those who are (morbidly) obese.
- Older adults patients, patients in emergency settings, or those on anticoagulation therapy who are at increased risk of complications from retro-muscular dissections.
- Patients with specific anatomical or surgical histories, such as recurrences after retro-muscular mesh placement, peritoneal resections, or prior IPUM repairs.
- Cases where robotic infrastructure or laparoscopic eTEP expertise is unavailable.

While IPUM+ provides improved outcomes for wound complications and morbidity in selected patients, it may not be the preferred technique for large hernias (>7 cm width) or defects close to bony structures. Preference would go for ventral TAPP, robotic assisted, procedures on a preperitoneal mesh reinforcement.

Additionally, although reoperation following LVHR is generally safe, surgeons should discuss with patients the potential long-term implications of intraperitoneal mesh placement, particularly its impact on future abdominal surgeries. In a small percentage of cases, unpredictable adhesions may arise. These can often be managed through safe lateral laparoscopic access followed by adhesiolysis before proceeding with elective surgery [4, 29].

### Practical Guidelines for Optimizing IPUM+ Outcomes

Preventive measures, including the use of adhesion barriers and meticulous surgical techniques, are essential to minimizing risks. Key practices include limited fixation devices, appropriate defect closure, and careful mesh selection. In cases involving uncomplicated mesh-related adhesions, modern management strategies such as minimally invasive adhesiolysis are effective for treating adhesions without necessitating mesh removal or additional major surgeries [4, 29].

#### Core Recommendations for IPUM+ Success

1. Mesh Selection: Use composite, macroporous, anti-adhesive mesh barriers, ensuring careful handling to preserve the protective layer.
2. Mesh Size: Tailor mesh size to the defect dimensions, patient BMI, and expected abdominal wall forces.
3. Defect Closure: Perform primary fascial closure for defects under 7 cm in width, maintaining physiological tension on the linea alba with non-resorbable monofilament sutures. This improves aesthetics, reduces dead space, and prevents skin bulging. For larger defects (typically exceeding 6 cm), dynamic closure techniques such as laparoscopic intracorporeal rectus aponeuroplasty (LIRA) or hybrid approaches may be more appropriate. These methods, by offloading tension, facilitate closure of larger defects. Hybrid techniques may include external open fascial closure with sac resection, and have shown to yield improved outcomes [28, 37].
4. Landing Zone Preparation: Excision of fatty tissue and preparation of the peritoneum are necessary to ensure secure fixation of the mesh to healthy closed fascial layers. This also promotes even tissue integration, as described by the Rives-Stoppa principles.
5. Mesh Coverage: Ensure complete coverage of the closed defect, with a minimum mesh overlap of 6 cm on all borders, unless contraindicated.
6. Fixation Strategy: Employ smoothly tied, non-resorbable cardinal trans fascial sutures to anchor the mesh permanently, especially in obese or recurrent cases, under controlled low intra-abdominal pressure.

## Conclusion: The Case for a Tailored Approach

### Evidential Defense of IPUM+

While historical concerns regarding intraperitoneal mesh persist, advancements in mesh design and surgical techniques have significantly enhanced the safety and efficacy of IPUM+. Current evidence supports its continued role in selected ventral hernia repairs, with benefits that outweigh the risks of mesh-related adhesions, particularly in patients for whom eTEP, ventral TAPP, or robotic techniques may not be suitable. Additionally, not all surgeons are trained in eTEP techniques or have access to robotic systems, making IPUM+ a practical and valid alternative for many settings.

### Emphasizing Individualized Treatment

A well-performed IPUM+ remains a competitive “toolbox” option for ventral hernia repair, particularly in obese patients, small- to moderate-sized hernias, or recurrent cases. It offers excellent long-term outcomes when applied in tailored cases and performed by skilled surgeons exercising good judgement.

The debate should not center on abandoning IPUM+, but rather on defining its role within a comprehensive, patient-centered approach to hernia repair. As evidence evolves, surgeons are encouraged to avoid overtreatment of small- to moderate-sized hernias and consider emerging hybrid IPUM+, ventral TAPP, robotic or eTEP techniques combining Transverse Abdominal Release (TAR) for larger sized hernia and open surgery for more complex cases.
